# Гипогонадотропный гипогонадизм вследствие патогенного варианта в гене POLR3B

**DOI:** 10.14341/probl13474

**Published:** 2026-01-18

**Authors:** О. А. Малиевский, Р. И. Малиевская, Е. В. Сайфуллина

**Affiliations:** Башкирский государственный медицинский университетРоссия; Bashkir State Medical UniversityRussian Federation

**Keywords:** дети, гипогонадотропный гипогонадизм, ген POLR3, синдром 4H, children, hypogonadotropic hypogonadism, POLR3 gene, 4H syndrome

## Abstract

Врожденный гипогонадотропный гипогонадизм (ВГГ) — группа заболеваний, вызванных нарушением синтеза или секреции гонадотропин-рилизинг-гормона (ГнРГ) и гонадотропных гормонов. В настоящее время описано более 20 генов, участвующих в развитии ВГГ. В структуре ВГГ наиболее часто встречаются формы заболевания, обусловленные патогенными вариантами в генах, участвующих в онтогенезе, миграции и выживании нейронов ГнРГ, тогда как патология генов, участвующих в действии/передаче сигналов ГнРГ в нормально развитых нейронах ГнРГ, встречается реже. В данной статье приведено первое в России описание редкой формы ВГГ в результате патогенных вариантов в гене POLR3B, встречающейся в 1,1% случаев ВГГ, являющейся компонентом гипомиелинизирующей лейкодистрофии 4Н и включающей в себя гипомиелинизацию, ВГГ, гиподонтию. Идентификация генетической природы заболевания у данной пациентки позволило не только установить причину ВГГ, но и диагностировать коморбидные состояния.

## Abstract (en)

Congenital hypogonadotropic hypogonadism (СНH) is a group of diseases caused by impaired synthesis or secretion of gonadotropin-releasing hormone (GnRH) and gonadotropin hormones. At present, more than twenty genes involved in the development of СНН have been described. In the structure of HGH, the most common forms of the disease are caused by pathogenic variants in genes involved in the ontogenesis, migration and survival of GnRH neurons, whereas pathology of genes involved in the action/transmission of GnRH signals in normally developed GnRH neurons is less common. This article describes a rare variant of СНН as a result of pathogenic variants in the POLR3B gene, occurring in 1.1% of cases of СНН, which is a component of hypomyelinating leukodystrophy 4H and includes hypomyelination, CHН, hypodontia. Identification of the genetic nature of the disease in this patient made it possible not only to establish the cause of CНН, but also to diagnose comorbid conditions.

## АКТУАЛЬНОСТЬ

Врожденный гипогонадотропный гипогонадизм (ВГГ) — гетерогенная группа заболеваний, вызванных нарушением синтеза или секреции гонадотропин-рилизинг-гормона (ГнРГ) и гонадотропных гормонов и характеризующихся задержкой полового развития и бесплодием [1]. Частота встречаемости ВГГ составляет 1:125 000 у лиц женского пола и 1:30 000 — у лиц мужского пола [2]. Примерно в половине случаев ВГГ сопровождается аносмией (синдром Каллмана) [3].

Гены, мутации в которых приводят к развитию изолированного ВГГ, можно разделить на две основные группы, в зависимости от биологической функции в системе нейронов ГнРГ. Первую группу составляют гены, участвующие в онтогенезе, миграции и выживании нейронов ГнРГ (AMH/AMHR2, ANOS1, CCDC141, CHL1, DCC/NTN1, FEZF1, HS6ST1, IGSF10, GLI3, NDNF, NRP1/NRP2, NSMF, PLXNA1/PLXNA3, CHD7, FGF8/FGFR1, FGF17, SEMA3А/SEMA3F, SEMA7A, SEMA3E, SOX10, SMCHD1, TCF12, TUBB3, PROKR2, AXL, DUSP6, SPRY4). Во вторую группу входят гены, участвующие в действии/передаче сигналов ГнРГ в нормально развитых нейронах ГнРГ: DMXL2, GNRH1, KISS1, KISS1R, KLB, LEP/LEPR, NROB1, PCSK1, TAC3, TACR3, POLR3A, POLR3B [4]. В структуре ВГГ наиболее часто встречаются формы заболевания, обусловленные патогенными вариантами в генах первой группы, что было продемонстрировано и в результате российского исследования: у 60,9% пациентов с ВГГ были выявлены мутации в генах, ответственных за развитие и миграцию ГнРГ-нейронов [5].

Врожденный гипогонадотропный гипогонадизм может быть клинически изолированным или сочетаться с гипо- или аносмией (синдром Каллмана), а также быть одним из признаков мультисистемных заболеваний/синдромов: септооптической дисплазии, CHARGE-синдрома, DAX-синдрома, Коффина-Сириса, лейкодистрофии и других состояний, клинические проявления которых включают нарушение слуха, зрения, когнитивных способностей, эпилепсию, расщелину неба, гипо- или олигодентию, пороки развития внутренних органов, надпочечниковую недостаточность [1][6–9].

## ОПИСАНИЕ СЛУЧАЯ

Пациентка А. направлена на консультацию детского эндокринолога для уточнения причины аменореи в возрасте 17 лет.

Анамнез жизни. Девочка от второй беременности, протекавшей с угрозой прерывания на пятом месяце из-за тонкой плаценты, первых родов на сроке 38 недель путем кесарева сечения из-за неоткрытия шейки матки. При рождении длина тела — 56 см (+3,76 SDS), вес — 3250 г (+0,36 SDS). На грудном вскармливании — до 5 месяцев. Голову уверенно удерживает с 2 месяцев, садится с 6 месяцев, ходит с 11 месяцев. Нервно-психическое развитие также протекало в соответствии с возрастом. В 5 месяцев появился первый зуб, затем до года зубов не было. Не было зачатка верхнего молочного клыка, что было подтверждено рентгенологическим исследованием. Смена молочных зубов — с 6–7 лет. В настоящее время все коренные зубы имеются. С 12-летнего возраста пациентка наблюдается у офтальмолога в связи с миопией (в настоящее время на фоне использования ночных корректирующих линз миопия OU (-7,25) не прогрессирует). Из анамнеза также известно, что пациентка является единственной дочерью у своих родителей, подобного заболевания в семье не было, отец пациентки умер от заболевания печени.

Анамнез заболевания. Впервые пациентка обратилась к гинекологу в возрасте 16 лет по поводу отсутствия менструаций. На момент обращения рост составлял 150 см (SDS=-2,03), вес — 52,3 кг, ИМТ — 23 кг/м² (SDS=0,88). Половое развитие соответствовало стадии В 3, Р 3. Выполнены исследования: 1) УЗИ органов малого таза. Размеры матки 14х12х8 мм. М-эхо — 0,9 мм, длина шейки матки — 15 мм, ширина — 11 мм, передне-задний размер — 6 мм. Правый яичник 22х12х13 мм, объем — 1,8 см³, гипоэхогенный, единичные фолликулы диаметром до 2 мм, левый яичник 13х6х9 мм, объем — 0,4 см³, гипоэхогенный, единичные фолликулы диаметром до 2 мм; 2) гормональный анализ крови: ФСГ — 5,77 мМЕ/мл (1,5–12,8 мМЕ/мл), ЛГ — 2,0 мМЕ/мл (2,0–6,3 мМЕ/мл), эстрадиол — 11,34 пмоль/л (50–220 пмоль/л), ТТГ — 3,292 мкМЕ/мл, свТ4 — 13,22 пмоль/л 3) кариотип — 46,ХХ. Пациентке диагностирована первичная аменорея. Проба с аналогом ГнРГ не проведена. Начата терапия 0,1% трансдермальным гелем эстрадиола гемигидрата. В последующем добавлен пероральный прием дидрогестерона с 14 по 28 день менструального цикла, который пациентка принимала в течение 7 мес, после чего была переведена на лечение комбинированным препаратом эстрадиола 2 мг/дидрогестерона 10 мг.

На фоне данной схемы лечения на УЗИ органов малого таза увеличились размеры: матки — до 29х30х19 мм, М-эхо — до 3,6 мм, шейки матки: длина — до 24 мм, ширина — до 19 мм, передне-задний — до 23 мм. Правый яичник 24х14х16 мм, объем — 2,8 см³, единичные фолликулы диаметром 3–5 мм (до 2 антральных фолликула в одном срезе). Левый яичник 21х13х12 мм, объем — 1,7 см³, единичные фолликулы диаметром 2–4 мм.

Была рекомендована консультация эндокринолога для исключения эндокринной причины аменореи.

При сборе анамнеза установлено, что у мамы и тети по маминой линии менструации с 16–17 лет. У мамы миопия, нет зачатков зубов мудрости. Наследственность по другой эндокринной патологии не отягощена.

## Результаты физикального, лабораторного и инструментального исследований

Рост — 160 см (SDS=-0,76), вес — 57 кг, ИМТ — 22 кг/м² (SDS=0,47). Скорость роста — 10 см/год. Пропорционального телосложения, удовлетворительного питания. Кожа физиологической окраски, нормальной влажности. Отмечаются густые ресницы, рост в 3 ряда. АД — 90/60 мм рт.ст. Щитовидная железа не увеличена. Половое развитие (на фоне проведенной заместительной гормональной терапии): В 4, Р 4.

На фоне отмены заместительной гормональной терапии в течение двух месяцев проведена проба с аналогом ГнРГ. Уровень ЛГ исходно 0, через 1 и 4 часа соответственно 0,12 и 0,12 мМЕ/мл. Уровень ФСГ исходно 0,22 мМЕ/мл, через 1 и 4 часа соответственно 0,421 и 0,36 мМЕ/мл. Уровень эстрадиола составлял 9,2 пмоль/л (80–330 пмоль/л). На основании данных результатов диагностирован гипогонадотропный гипогонадизм.

В рамках программы «АльфаЭндо» в ФГБУ «НМИЦ эндокринологии» Минздрава России проведено исследование ДНК пациентки методом массового параллельного секвенирования (панель «Гипогонадотропный гипогонадизм»).> В гене POLR3B (NM_018082.6) обнаружены ранее описанные в литературе патогенные варианты: c.1568T>A и c.2084-6A>G в гетерозиготном состоянии. По результатам автоматического секвенирования по Сэнгеру у пациентки подтверждено наличие обоих вариантов, у ее матери — только одного варианта c.1568T>A в гетерозиготном состоянии, что позволило сделать вывод о компаунд-гетерозиготном состоянии вариантов c.1568T>A и c.2084-6A>G в гене POLR3B у пациентки.

Биаллельные мутации в гене POLR3B описаны при гипогонадотропном гипогонадизме и/или гипомиелинизирующей лейкодистрофии с олигодонтией или без нее (#614381) с аутосомно-рецессивным типом наследования.

С учетом литературных данных о развитии лейкодистрофии при биаллельных мутациях в гене POLR3B проведена оценка неврологического статуса пациентки и выполнена МРТ головного мозга. При отсутствии жалоб у пациентки обнаружены окуломоторные нарушения: отсутствие плавности следящих движений глазных яблок, билатеральный горизонтальный нистагм и легкая статическая сенситивная атаксия, двигательных, рефлекторных нарушений в конечностях не выявлено. По данным МРТ головного мозга выявлено симметричное поражение перивентрикулярного и юкстакортикального белого вещества лобных, теменных, височных долей, гипотрофия мозжечка, уменьшения размеров гипофиза (сагиттальный размер гипофиза 8 мм, вертикальный 3–4 мм, фронтальный 12 мм) (рис. 1).

**Figure:**
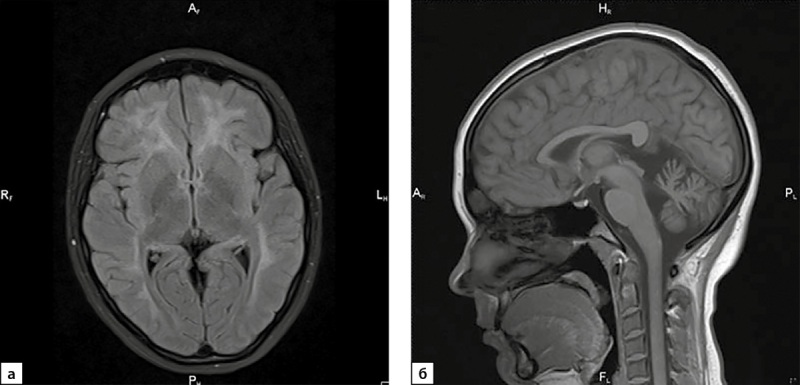
Рисунок 1. МРТ головного мозга пациентки с POLR3-ассоциированной лейкодистрофией (а — axial, Т2 FLAIR; б — saggital, T1).

## ОБСУЖДЕНИЕ

Представлено первое в России описание одной из редких форм ВГГ вследствие патогенных биаллельных вариантов в гене POLR3B. По зарубежным литературным данным, данная форма встречается всего у 0,7–1,1% пациентов с гипогонадотропным гипогонадизмом [10][11]. ВГГ развивается в результате нарушений секреции или действия ГнРГ, точный механизм развития которых до конца не ясен. Можно предположить, что причиной является нарушение двунаправленной связи между аксоном и миелином, когда повреждение миелина вызывает дегенерацию аксонов, а дегенерация аксонов приводит к потере миелина [12]. Вероятно, данным механизмом можно объяснить снижение базальных уровней ЛГ и ФСГ в динамике, а также низкий гормональный ответ на стимуляцию с аналогом ГнРГ у представленной пациентки.

Особенностью данного заболевания является сочетание гипогонадизма с гипомиелинизирующей лейкодистрофией. Гипомиелинизирующие лейкодистрофии — группа генетически гетерогенных заболеваний, характеризующихся нарушением образования миелина центральной нервной системы. Известно несколько десятков генов, патогенные мутации в которых приводят к развитию этих заболеваний (https://www.omim.org/entry/312080, дата обращения 03.05.2024). Биаллельные патогенные мутации в генах POLR3B, POLR3А, а также POLR1C приводят к развитию лейкодистрофии, ассоциированной с РНК-полимеразой III (Pol III), которая характеризуется гипомиелинизацией, гипо- или олигодентией и гипогонадотропным гипогонадизмом (hypomyelination, hypodontia, hypogonadotropic hypogonadism, 4H лейкодистрофия) [13].

РНК-полимераза III транскрибирует небольшие нетранслируемые РНК, участвующие в регуляции основных клеточных процессов (транскрипции, процессинга РНК и трансляции), необходимых для синтеза полноценных белков миелина центральной нервной системы [14]. Фермент состоит из 17 субъединиц. Ген POLR3B кодирует вторую по величине субъединицу Pol III, которая совместно с самой крупной субъединицей (POLR3А), кодируемой геном POLR3А, составляют каталитический центр фермента. Механизм влияния сниженной функции Pol III на олигодендроциты, изучен недостаточно, но в исследовании Macintosh J. et al. (2023) показано, что снижение экспрессии фермента изменяло скорость пролиферации клеток-предшественников олигодендроцитов и нарушало дифференцировку клеток-предшественников в зрелые олигодендроциты [15].

POLR3B — ассоциированная лейкодистрофия преобладает среди пациентов с 4H лейкодистрофией, что было показано в двух исследованиях. В международном обсервационном исследовании 105 пациентов с молекулярно-генетически подтвержденными случаями болезни: у 43 пациентов заболевание было обусловлено мутациями в гене POLR3A и у 62 пациентов — в гене POLR3B. Отмечено, что большинство из этих пациентов имели европейское происхождение (53 человека из 62) [13]. По результатам многоцентрового ретроспективного исследования с участием 150 пациентов с 4H лейкодистрофией показано, что у 37,3% пациентов причиной заболевания явились патогенные мутации в гене POLR3A, у 54% — в гене POLR3B и у 8,7% — в гене POLR1C [16].

4H лейкодистрофия характеризуется клиническим полиморфизмом: по возрасту дебюта заболевания, спектру и степени выраженности симптомов болезни. Возраст начала заболевания обычно варьирует от младенчества до детства, но в ряде случаев, как и у наблюдаемой нами пациентки, симптомы болезни могут возникнуть в позднем подростковом или раннем взрослом возрасте. Гипомиелинизация является обязательным проявлением заболевания, несмотря на некоторые различия по степени и частоте вовлеченности участков белого вещества, в том числе мозжечка, мозолистого тела у пациентов с мутациями в генах POLR3B и POLR3A [13]. Дебют заболевания у большинства пациентов начинается с неврологической симптоматики: задержка развития, мозжечковые симптомы: интенционный тремор, дисметрия, а также нарушения следящих движений глазных яблок и нистагм. Пирамидные и экстрапирамидные нарушения могут развиваться позднее, приводя к потере амбулаторности в конце первого — в начале второго десятилетия жизни. Когнитивные способности варьируются от нормальных до трудностей с обучением или от легкой до умеренной умственной отсталости (наиболее часто), с медленным ухудшением состояния. У наблюдаемой нами пациентки, несмотря на отсутствие жалоб, выявлены окуломоторные нарушения, нистагм и легкая атаксия. Основным клиническим проявлением болезни у нее явился гипогонадотропный гипогонадизм.

К другим проявлениям данного синдрома у нашей пациентки относилось отсутствие зачатка верхнего молочного клыка. Зубные аномалии при данном синдроме характеризуются широким спектром: описано наличие натальных зубов, задержка прорезывания зубов с неправильным порядком прорезывания молочных зубов, гиподонтия и олигодонтия [13].

У 87% пациентов отмечалась близорукость, часто выраженная и обычно прогрессирующая, что также отмечалось и у нашей пациентки. Помимо близорукости у пациентов (пожилых) описана атрофия зрительного нерва, иногда развивалась катаракта. Низкий рост описан у половины пациентов, что чаще наблюдалось у детей с дебютом заболевания в возрасте до 3 лет [13]. Учитывая нормальный рост и высокую скорость роста у нашей пациентки, исследование уровня инсулиноподбного фактора роста 1 и стимулированной секреции гормона роста не проводилось.

В гене POLR3B описано более 70 патогенных и вероятно патогенных вариантов: большинство пациентов являются компаунд-гетерозиготами (https://www.ncbi.nlm.nih.gov/clinvar, дата обращения 01.06.2024). Возраст манифестации клинических проявлений болезни варьировал от дебюта внегонадных проявлений в раннем возрасте до появления первых признаков в виде задержки полового развития, как произошло у нашей пациентки [13]. Мягкое течение заболевания в описываемом нами случае обусловлено вариантом c.1568T>A (p.Val523Glu), наличие которого в гомозиготном или компаунд-гетерозиготном состоянии чаще приводит к подобному фенотипу [13].

## ЗАКЛЮЧЕНИЕ

Описанный случай демонстрирует недостаточную диагностику ВГГ у девушек, обусловленную возможностью спонтанного телархе, а также низкой осведомленностью врачей-гинекологов о современных возможностях диагностики ВГГ, в том числе о методах молекулярно-генетического исследования. Идентификация генетической природы различных вариантов ВГГ позволяет не только подтвердить диагноз и установить причину гипогонадизма, но и диагностировать и прогнозировать течение коморбидных состояний.

## ДОПОЛНИТЕЛЬНАЯ ИНФОРМАЦИЯ

Источники финансирования. Работа выполнена по инициативе авторов без привлечения финансирования.

Конфликт интересов. Авторы декларируют отсутствие явных и потенциальных конфликтов интересов, связанных с содержанием настоящей статьи.

Участие авторов. Все авторы внесли существенный вклад в получение, анализ и интерпретацию результатов, поиск и оценку данных литературы, написание статьи или внесение в рукопись существенных (важных) правок с целью повышения научной ценности статьи. Все авторы одобрили финальную версию статьи перед публикацией, выразили согласие нести ответственность за все аспекты работы, подразумевающую надлежащее изучение и решение вопросов, связанных с точностью или добросовестностью любой части работы.

Согласие пациента. Пациентка добровольно подписала информированное согласие на публикацию персональной медицинской информации в обезличенной форме в журнале «Проблемы эндокринологии».
